# Identification and Functional Analysis of Epigenetically Silenced MicroRNAs in Colorectal Cancer Cells

**DOI:** 10.1371/journal.pone.0020628

**Published:** 2011-06-16

**Authors:** Hongli Yan, Ae-jin Choi, Byron H. Lee, Angela H. Ting

**Affiliations:** 1 Genomic Medicine Institute, Lerner Research Institute, Cleveland Clinic Foundation, Cleveland, Ohio, United States of America; 2 Glickman Urological and Kidney Institute, Cleveland Clinic Foundation, Cleveland, Ohio, United States of America; Ludwig-Maximilians-Universität München, Germany

## Abstract

Abnormal microRNA (miRNA) expression has been linked to the development and progression of several human cancers, and such dysregulation can result from aberrant DNA methylation. While a small number of miRNAs is known to be regulated by DNA methylation, we postulated that such epigenetic regulation is more prevalent. By combining MBD-isolated Genome Sequencing (MiGS) to evaluate genome-wide DNA methylation patterns and microarray analysis to determine miRNA expression levels, we systematically searched for candidate miRNAs regulated by DNA methylation in colorectal cancer cell lines. We found 64 miRNAs to be robustly methylated in HCT116 cells; eighteen of them were located in imprinting regions or already reported to be regulated by DNA methylation. For the remaining 46 miRNAs, expression levels of 18 were consistent with their DNA methylation status. Finally, 8 miRNAs were up-regulated by 5-aza-2′-deoxycytidine treatment and identified to be novel miRNAs regulated by DNA methylation. Moreover, we demonstrated the functional relevance of these epigenetically silenced miRNAs by ectopically expressing select candidates, which resulted in inhibition of growth and migration of cancer cells. In addition to reporting these findings, our study also provides a reliable, systematic strategy to identify DNA methylation-regulated miRNAs by combining DNA methylation profiles and expression data.

## Introduction

MicroRNAs (miRNAs) are small, non-coding RNAs that regulate gene expression and play pivotal roles in normal cellular processes including proliferation, differentiation, and apoptosis [1]. Both aberrant expression and silencing of miRNAs have been observed in human cancers, suggesting potential oncogenic and tumor suppressor functions for these miRNAs [2], [3]. The biogenesis of miRNAs involves transcription of a long primary transcript (pri-miRNA) by RNA polymerase II [4], cleavage into an intermediate product (pre-miRNA) by Drosha [5], and final processing into the mature miRNA by Dicer [6]. Each step of the process is highly regulated, and dysregulation at any level can result in inappropriate miRNA functions. Of particular interest to our study is miRNA transcriptional regulation by DNA methylation. Generally, gene promoter DNA methylation is negatively correlated to gene expression and can account for aberrant tumor suppressor gene silencing in a variety of human cancers [7]. Similar to these POLR2A transcribed protein coding genes, miRNA transcription may also be silenced by promoter DNA methylation.

Epigenetically silenced miRNAs have been discovered in cancers based on differential expression between normal tissues and tumors or between baseline and DNA demethylated cancer cells. For instance, Bandres *et al.* first identified 23 miRNAs that are down-regulated in primary colorectal cancers compared with matched normal colorectal epithelium and subsequently discovered that miR-129-2, miR-9-1, and miR-137 are silenced by DNA methylation in cancer [8]. Toyota *et al.* treated HCT116 colorectal cancer cells with the demethylating agent, 5-aza-2′-deoxycytidine (5-aza-dC), and compared miRNA expression profiles between the treated and the untreated cells to identify silencing of miR-34b/c by promoter DNA hypermethylation [9]. Furthermore, Lujambio *et al.* compared the miRNA expression profile of wildtype HCT116 cells with that of its demethylated isogenic derivative, DNA methyltransferase −1 and −3b Double Knockout (DKO) cells, and found 18 miRNAs up-regulated in DKO cells [10]. Subsequently, they confirmed that DNA methylation is responsible for the silencing of miR-124a in colon cancer.

A literature search using MEDLINE revealed that 16 miRNAs are known to be epigenetically silenced by DNA methylation [8], [9], [10], [11], [12], [13], [14], [15], [16], [17]. DNA methylation refers to the covalent addition of a methyl group to the 5-position of cytosines, usually in a CpG dinucleotide context in mammalian differentiated cells [18]. Genomic regions with a high density of CpGs are termed CpG islands and are often the sites of regulatory DNA methylation. Considering that 16% of the annotated human miRNAs are located within 1000 bp of a CpG island [19], we surmise that epigenetic regulation of miRNAs might be more common than reported thus far. Previous studies relied on differential expression of miRNAs to identify candidates for subsequent epigenetic evaluation. This approach introduces bias that limits the number of epigenetically regulated miRNAs that can be discovered. For example, tissue-specific miRNAs that are regulated by DNA methylation may be equivalently methylated in normal tissues and tumors, resulting in a lack of differential expression between normal and cancerous specimens. Furthermore, miRNAs with low expression levels cannot be reliably identified because the differences in expression between normal and cancer or between baseline and demethylated conditions will likely fall below conventional cutoffs (between 2 and 1.5 fold). Finally, residual methylation persists in both pharmacologically and genetically demethylated cells [20]. Such methylation may be critical in maintaining cell viability, potentially through persistent repression of key miRNAs. This would result in such miRNAs not being identified through expression-based strategies.

To overcome the discovery bias introduced by expression-based identification strategy, we directly utilized global DNA methylation patterns to identify miRNAs regulated by DNA methylation in HCT116 and DKO colon cancer cells. We have previously mapped genome-wide DNA methylation in these cell lines using methyl CpG binding domain (MBD)-isolated Genome Sequencing (MiGS) [21]. We first identified miRNAs with proximal DNA methylation as candidates. We then cross-referenced the list of candidates with miRNA expression data as supporting evidence. Using this approach, we successfully identified both known and novel DNA methylation-regulated miRNAs. Here we provide a more comprehensive catalogue of epigenetically regulated miRNAs in colon cancer cells, demonstrating that epigenetic regulation of miRNAs is prevalent in these cancer cell lines. Furthermore, functional studies of select miRNAs provide biological relevance for such epigenetic regulation in the context of colon cancer.

## Results

### Candidate miRNAs regulated by DNA methylation were identified by proximal genomic DNA methylation and supporting expression data

**Figure 1:**
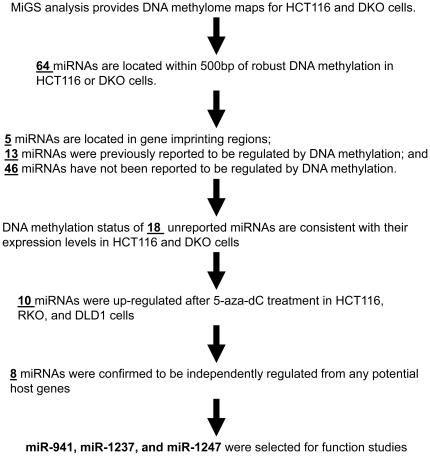
Overview of the screening approach performed in this study.

We previously used MiGS to construct genome-wide DNA methylation profiles for the HCT116 cell line and its demethylated isogenic cell line, DKO [21]. DKO cells are HCT116 cells deleted for DNA methyltransferase −1 and −3b and retain <5% genomic DNA methylation [20]. DNA methylated fractions of the genome were isolated by incubation with recombinant MBD proteins, sequenced on an Illumina GAII sequencer, and mapped back to the reference genome to generate individual methylome profiles. To identify DNA methylation-regulated miRNAs, we searched for miRNAs with evidence of DNA methylation within 500 bp of their annotated positions in HCT116 and DKO cells. Using this approach, we identified 64 candidate miRNAs for subsequent analysis (Figure 1).

**Figure 2:**
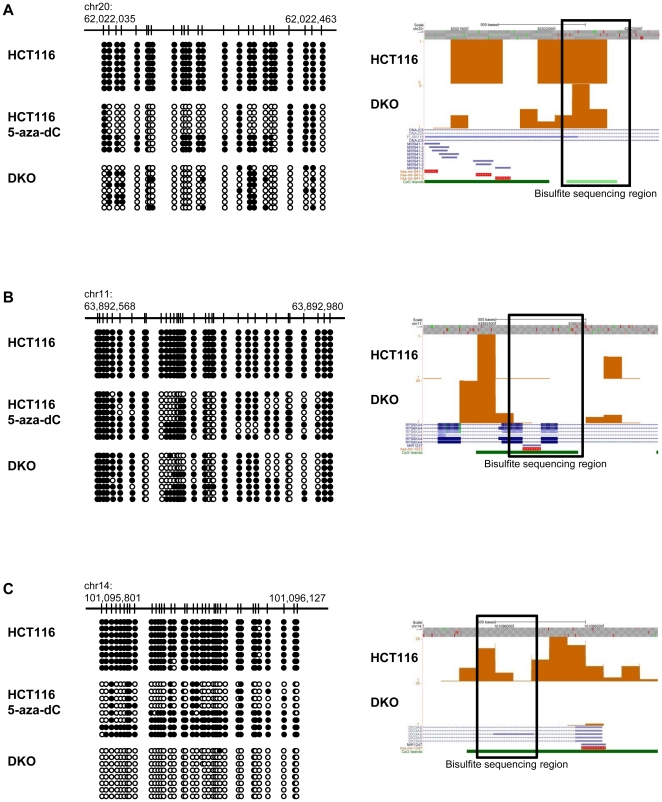
Representative bisulfite sequencing of candidate miRNAs. Bisulfite sequencing was carried out for (**A**) miR-941, (**B**) miR-1237, and (**C**) miR-1247 in parental HCT116 cells, HCT116 cells treated with 5 µM 5-aza-dC for 48 hr, and DKO cells. The UCSC genome browser screen capture shows the DNA methylation signal in each sample. The rectangles mark the regions validated by bisulfite sequencing. Each circle represents a CpG dinucleotide. Black circles represent methylated cytosines while white circles represent unmethylated cytosines.

Of the 64 candidate miRNAs, 13 have been reported to be regulated by DNA methylation (Table S1). Another 5 belong to a large miRNA cluster located in the human DLK1–GTL2 imprinted domain at 14q32 and are silenced on the paternal chromosome by DNA methylation [22]. Therefore, we focused on the remaining 46 miRNAs that have not been reported to be regulated by DNA methylation for detailed validation. First, we randomly selected 6 miRNAs for bisulfite sequencing analysis to verify the DNA methylation status detected by MiGS (Figure 2 and Figure S1). The bisulfite sequencing data corroborated the MiGS data at all 6 loci.

Next, we reasoned that loss of DNA methylation in DKO cells when compared to HCT116 cells should result in increased expression of the candidate miRNAs while retention of DNA methylation should correlate to persistent silencing of the candidate miRNAs. Therefore, we examined the expression microarray data for the 46 candidates in HCT116 and DKO cells (Table S2). Using 1.5-fold change as our threshold, we identified 18 miRNAs as having consistent expression data with their DNA methylation status in HCT116 and DKO cells (Table S1). We performed miRNA-specific quantitative real-time RT–PCR (miR-qRT–PCR) analysis for 6 select miRNAs to verify the expression data determined by the microarray (Figure S2). The miR-qRT-PCR results confirmed the microarray data for the 6 miRNAs, which included both known and candidate DNA methylation-regulated miRNAs. Therefore, based on empirical genomic DNA methylation and supporting microarray expression data, we identified 18 novel candidate miRNAs that may be transcriptionally regulated by DNA methylation.

### Candidate miRNAs re-express after 5-aza-2′-deoxycytidine treatment

**Figure 3:**
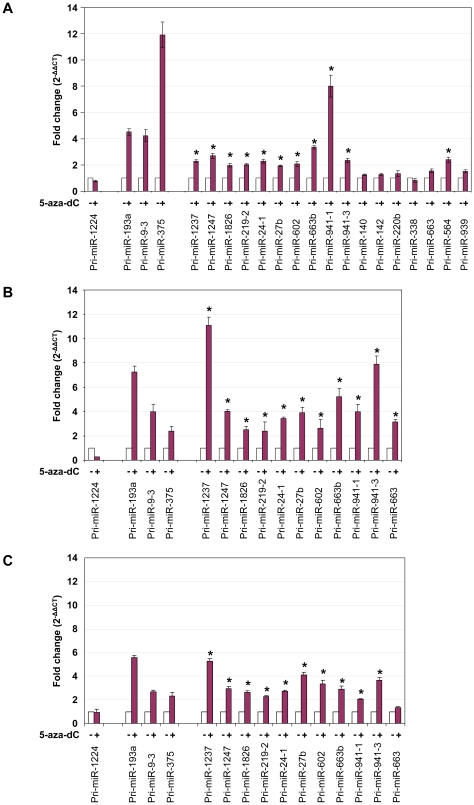
Expression analysis of candidate miRNAs after 5-aza-dC treatment. Real-time RT-PCR analysis was carried out to assess candidate primary miRNAs (Pri-miRNAs) levels in (**A**) HCT116, (**B**) RKO, and (**C**) DLD1 cells before and after treatment with 5 µM 5-aza-dC for 48 hr. miR-1224 was included as a negative control. miR-193a, miR-375, and miR-9-3 were included as positive controls. * indicates significant increase in Pri-miRNA expression after 5-aza-dC treatment (p<0.05). Expressions of miRNAs were internally normalized to the expression levels of *GAPDH*, and normalized expression for each miRNA before 5-aza-dC treatments was set to 1.

To validate that the candidate miRNAs were indeed regulated by DNA methylation, we treated HCT116, RKO, and DLD1 colon cancer cells with the demethylating agent 5-aza-2′-deoxycytidine (5-aza-dC) to determine whether these miRNAs are re-expressed after 5-aza-dC treatment. As DNA methylation regulates gene expression at the transcriptional level, we assayed for the expression of the primary miRNAs (Pri-miRNA) by real time RT-PCR [23]. included Pri-miR-193a, −9-3, and −375, which were known to be transcriptionally regulated by DNA methylation in HCT116 cells [8], as positive controls and Pri-miR-1224, which was demethylated but remained silenced in DKO cells in this study, as a negative control. We tested 17 out of the 18 novel candidates because none of the primers designed for miR-1234 yielded successful PCR products. In HCT116 cells, we found 11 out of 17 miRNAs to be significantly up-regulated after 5-aza-dC treatment (Figure 3A). We further independently confirmed all 11 candidates in RKO cells (Figure 3B) and 10 out of the 11 candidates in DLD1 cells (Figure 3C). To verify that the up-regulation of these miRNAs was consequent to DNA demethylation after 5-aza-dC treatment, we assessed changes in DNA methylation status in the proximal regions of miR-941-1/3, miR-1237 and miR-1247 by bisulfite genomic sequencing (Figure 2). Demethylation was observed at these loci in HCT116 cells treated with 5-aza-dC, supporting that the re-expression observed was due to DNA demethylation.

Finally, intronic miRNAs can be co-transcribed from host gene promoters and therefore, are not independently regulated at the transcriptional level [24], [25]. Hence, to fully understand the significance of the DNA methylation we observed for our 10 candidate miRNAs, we examined the genomic contexts of the 10 candidates more closely (Table 1). miR-1247, miR-1826, and miR-219-2 are located in intergenic regions and therefore, are unlikely to be co-regulated by a host gene promoter. The remaining 7 candidates are located in introns of RefSeq or EST transcripts and were studied further. Based on the genome-wide DNA methylation profiles, none of the putative host genes, except for the non-coding RNA *ANKRD30BL*, have significant DNA methylation at their promoters (Table 1 and Figure S3), suggesting that these putative host genes are not regulated by promoter DNA methylation.

**Table 1:** Genomic context of candidate miRNAs.

miRNA	Genomic context	Putative host gene promoter methylation*	Host gene expression after 5-aza-dC treatment
hsa-miR-1237	*RPS6KA4* intron	No methylation	No change
hsa-miR-1247	Intergenic	N/A	N/A
hsa-miR-1826	Intergenic	N/A	N/A
hsa-miR-219-2	Intergenic	N/A	N/A
hsa-miR-24-1	*C9orf3* intron	No methylation	Inconsistent increase in expression
hsa-miR-27b	*C9orf3* intron	No methylation	Inconsistent increase in expression
hsa-miR-602	*AB058779* intron	No methylation	No change
hsa-miR-663b	*ANKRD30BL* intron	Methylation in HCT116	Not expressed
hsa-miR-941-1	*DNAJC5* intron	No methylation	No change
hsa-miR-941-3	*DNAJC5* intron	No methylation	No change

Note: * Putative host gene promoter is defined as ±1000 bp from the annotated transcription start sites.

**Figure 4:**
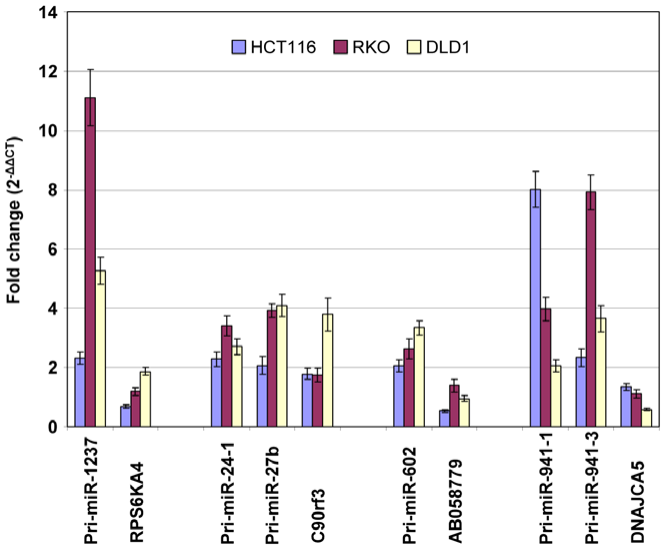
Expression analysis of putative host genes after 5-aza-dC treatment. Real-time RT-PCR analysis was carried out to assess putative host gene expression in response to 5-aza-dC treatments in HCT116 (blue), RKO (dark pink), and DLD1 (yellow). The changes in embedded miRNA expression were also plotted for side-by-side comparisons. All expressions were internally normalized to the expression levels of *GAPDH*, and normalized expression for each host gene and miRNA before 5-aza-dC treatments was set to 1.

Nonetheless, we postulated that if the putative host gene transcription dictates the expression of the embedded candidate miRNAs, we should detect increased host gene expression after 5-aza-dC treatment in HCT116, RKO, and DLD1 cells. Such increases in the host gene expression would correspond to the increases observed for the embedded miRNAs in these same cells after 5-aza-dC treatment. Conversely, if the putative host genes do not respond to 5-aza-dC treatment in the same manner as the embedded miRNAs, the miRNAs are likely transcribed independently from the putative host genes. In this latter scenario, DNA methylation we discovered near the miRNAs would be most meaningful in their transcriptional regulation. Based on qRT-PCR results, we determined that miR-1237, miR-602, miR-941-1, miR-941-3, and miR-663b are transcriptionally independent from their respective putative host genes (Figure 4). The expression of *ANKRD30BL*, the putative host gene for miR-663b, was not detectable before or after 5-aza-dC experiments and therefore, not plotted in Figure 4. For miR-24-1 and miR-27b, the data is less conclusive, and we could not rule out that these two mi-RNAs can be co-synthesized with the host gene, *C9orf3* [26]. Altogether, we identified and confirmed at least 8 novel miRNAs that are transcriptionally regulated by DNA methylation.

### miR-941 and miR-1247 suppress cell growth and migration in colon cancer cells

We proceeded to test the biological functions of these epigenetically regulated miRNAs to gain insights into the relevance of their DNA methylation in colon cancer cells. To determine if expression of these epigenetically silenced miRNAs would affect cancer cell growth and migration, we transfected HCT116 cells with miRNA mimics of miR-941 (the mature form of miR-941-1 and -3), miR-1237, miR-1247, and a non-coding negative control (AllStars Neg.).

**Figure 5:**
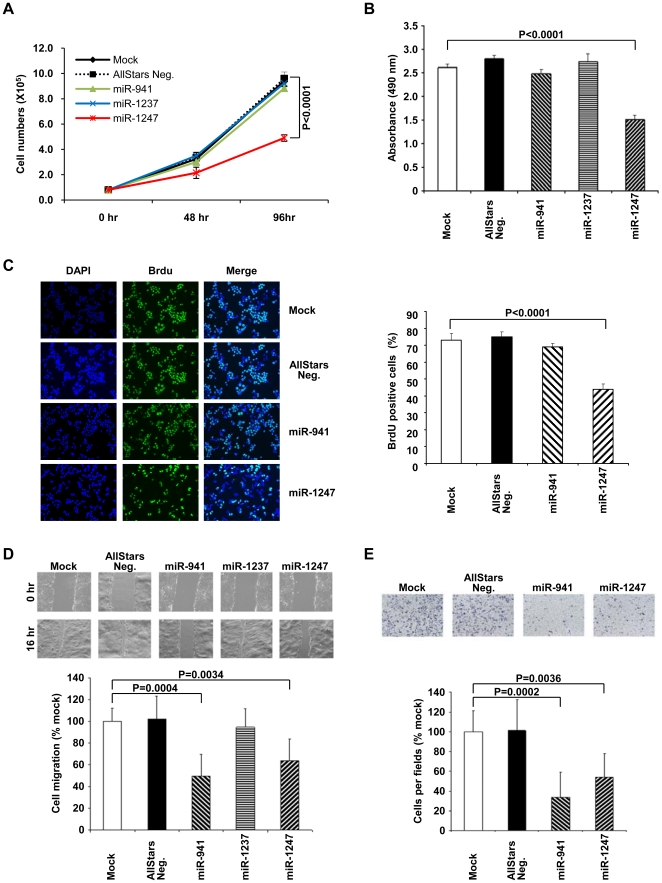
Functional analysis of miR-941, miR-1237, and miR-1247 in HCT116 cells. (**A**) Growth curves and (**B**) MTT assays of mock transfected HCT116 cells and HCT116 cells transfected with non-coding negative control miRNA (AllStars Neg.), miR-941, miR-1237, or miR-1247 mimics. (**C**) BrdU incorporation assay of mock transfected HCT116 cells and HCT116 cells transfected with non-coding negative control miRNA (AllStars Neg.), miR-941, or miR-1247. A representative field for each experimental condition is shown. The bar graph represents the average percentage (%) of BrdU positive cells. (**D**) Wound-healing assay for mock transfected HCT116 cells and cells transfected with negative control (AllStars Neg.), miR-941, miR-1237, or miR-1247 mimics. Photographs were taken immediately after wounding and 16 hr later. The results were quantified and normalized to the mock transfected cells. (**E**) Transwell migration assays for mock transfected HCT116 cells and cells transfected with negative control (AllStars Neg.), miR-941, or miR-1247 mimics. Representative fields of invasive cells on the membrane are shown. The bar graph represents the average number of cells on the underside of the membranes in each treatment normalized to mock transfected cells.

First, we performed growth curve analysis (Figure 5A) and MTT assay (Figure 5B) to assess cell growth and metabolic fitness. We found that ectopic expression of miR-1247 resulted in a significant decrease in HCT116 cell growth and metabolic activity as measured by the two assays respectively. BrdU incorporation assay independently verified that the decreased growth and metabolism is likely a consequence of decreased proliferation in these cells (Figure 5C). We observed a similar decrease in cell proliferation and metabolic function in DLD1 colon cancer cells transfected with miR-1247 mimic (Figure S4A and B). Conversely, in DKO cells, where miR-1247 is already demethylated and re-expressed, transfection with additional miR-1247 mimics did not have any effects on cell growth and metabolism (Figure S5A and B). These observations suggested that miR-1247 may be tumor-suppressive in colon cancer and its epigenetic silencing is therefore beneficial for cancer growth.

Furthermore, computationally predicted targets of miR-941 and miR-1247 included several genes involved in cell migration and invasion. For example, ADAM metallopeptidase domain 15 (*ADAM15*) is a predicted target of miR-1247 and encodes a transmembrane glycoprotein important for cell adhesion [27]. We postulated that down-regulation of these predicted targets by ectopic expression of miR-941 and miR-1247 would negatively impact cell migration. Hence, we tested the effects of ectopic expression of miR-941, miR-1237, and miR-1247 on cell mobility in HCT116 cells. In wound-healing assays, HCT116 cells transfected with miR-941 and miR-1247 mimics were significantly impaired in their ability to repopulate wounded areas when compared to mock, negative control, or miR-1237 transfected cells (Figure 5D). These observations were further confirmed using the transwell migration assay (Figure 5E). Finally, similar suppressive effects on cell migration were independently observed in DLD1 cells (Figure S4C) and DKO cells (Figure S5C) transfected with miR-941 and miR-1247 mimics. Taken together, these observations suggest that the epigenetic silencing of miR-941 and miR-1247 may facilitate colon cancer cell migration and invasion.

## Discussion

MicroRNAs are important regulatory molecules that modulate gene expression in both developmental and disease processes. Their regulation is, therefore, a critical component in understanding each biological context. Genomic DNA methylation has been shown to regulate the transcription of a handful of miRNAs in cancer. These previous studies aimed at identifying DNA methylation-regulated miRNAs relied on screening for differentially expressed miRNAs from cancer and normal comparisons or re-expression of miRNAs in DNA demethylated conditions. An expression-based approach introduces a discovery bias against equivalently methylated, therefore equivalently silenced, miRNAs in the comparison groups. This type of epigenetically regulated miRNA could still be important for cancer development. For instance, equivalently silenced miRNAs in normal and cancer may be significant for cancer in a tissue-specific manner. Furthermore, residual DNA methylation in induced demethylation conditions could sustain silencing of critical miRNAs, resulting in lack of detectable differential expression and failure to identify such epigenetically regulated miRNAs. Finally, miRNAs with marginal differential expression below typical thresholds would also be missed when the screening is based solely on differential expression.

To overcome the above biases and more comprehensively identify DNA methylation-regulated miRNAs in colon cancer, we screened for candidate miRNAs based on empirical evidence of genomic DNA methylation in HCT116 and DKO colon cancer cells. We systematically searched the DNA methylome maps generated by MiGS in these cells for miRNAs located within 500bp of DNA methylation signals. We found 64 candidates, including 5 known imprinted miRNAs, 13 known DNA methylation-regulated miRNAs, and 46 novel candidates. Subsequently, we confirmed that 8 novel candidates were indeed regulated by DNA methylation in HCT116, DKO, RKO, and DLD1 colon cancer cells. Together with the detection of known DNA-methylation regulated miRNAs, our study identified a total of 26 epigenetically regulated miRNAs, demonstrating that our approach is effective and efficient.

It is important to note that we may still be missing DNA methylation-regulated miRNAs derived from long primary transcripts. Previous studies focused on miRNAs with their stem-loop sequences embedded in or near CpG islands, and our current study focused on miRNAs with DNA methylation proximal to the stem-loop sequences. While these similar strategies allow for systematic screening, they likely underestimate the total number of miRNAs regulated by DNA methylation because transcription initiation for the primary miRNAs can be far upstream from the stem-loop sequence. For example, the primary transcript of miR-21 is 3433 nucleotide-long while the mature miR-21 is encoded by residues +2445 to +2516 in HeLa cells [28]. Recently, Chim *et al.* reported that miR-34a was regulated by promoter CpG island DNA methylation located >30 kb upstream from the mature miR-34a [29]. Therefore, more miRNAs may be regulated by promoter DNA methylation but will not be readily identified using a systematic approach that relies on the current genome annotation.

We further examined the biological relevance of the epigenetic silencing of the newly identified DNA methylation-regulated miRNAs. We focused on two important aspects of cancer development, growth and migration, and selected miR-941, miR-1237, and miR-1247 for functional studies. Ectopic expression of miR-1247 significantly reduced cancer cell proliferation and migration in HCT116 and DLD1 cells, suggesting that miR-1247 may function as a tumor suppressor. These observations are consistent with the functions of several predicted targets of miR-1247 (http://www.microrna.org). For example, Citron (CIT), a serine/threonine kinase, is present at the cleavage furrow and midbody during cytokinesis and is essential for cellular abscission [30], [31]. CIT also regulates the G2/M transition in rat hepatocytes [32], and knocking down CIT inhibited the proliferation of hepatocellular carcinoma cells [33]. Another potential target for miR-1247 is FosB, which dimerizes with Jun protein to activate transcription of proteases involved in tumor migration and invasion [34]. Finally, the transmembrane glycoprotein, ADAM15, might also be targeted by miR-1247, to facilitate prostate cancer metastasis [35]. These computationally predicted targets will need to be empirically validated in the future.

Interestingly, in DKO cells, where miR-1247 is demethylated and expressed at higher levels than HCT116 cells, additional exposure to miR-1247 mimics did not result in decreases of cell growth but caused impaired migration. The discrepancies between the phenotypes of ectopic miR-1247 expression in HCT116, DLD1, and DKO cells may reflect the different concentrations at which distinct cellular functions are modulated by miR-1247. Alternatively, it may be due to the presence of different targets for miR-1247 in HCT116 and DKO cells. These two possibilities will require additional studies to clarify.

While differentially methylated and expressed miRNAs are important for cancer development, we proposed that residual DNA methylation in demethylated conditions, such as in DKO cells, may also be critical. Our functional study of miR-941 substantiated this hypothesis. We found that over-expression of miR-941 was able to significantly inhibit cell migration in both HCT116 and DKO cells and additionally in DLD1 cells. This result is reasonable considering several high priority predicted targets of miR-941 are related to cell migration. For instance, matrix metallopeptidase 24 (MMP24) is a predicted target for miR-941, and it facilitates tissue remodeling and cell migration in endometrium from endometriosis patients [36]. Similar type of DNA methylation-regulated miRNAs would be missed by previous expression-based approach but could be readily captured by our study design. Six out of the 8 novel epigenetically regulated miRNAs identified in our study retained proximal DNA methylation in DKO cells, where <5% genomic DNA methylation is left compared to its parental HCT116 cells.

In summary, our approach to discover DNA methylation-regulated miRNAs yielded a number of previously undiscovered candidates. We have shown through ectopic expression and functional assays that at least two of these miRNAs may be particularly important in tumor progression. Our method is a useful complement to the traditional approach of using an expression-based scheme to identify novel miRNAs silenced by DNA methylation.

## Materials and Methods

### Cell culture and drug treatment

Colorectal cancer cell lines HCT116, DLD1, RKO and DKO cells [25] were cultured in McCoy's 5A media (Invitrogen, San Diego, CA) containing 10% fetal bovine serum (Gibco, Grand Island, NY) at 37°C in 5% CO_2_ atmosphere. Cells were harvested by scraping, and cell pellets were rinsed twice with PBS (Gibco, Grand island, NY). Total RNA was isolated using Trizol (Invitrogen, San Diego, CA) extraction according to manufacturer's instructions. Total RNA was also treated with DNase I (Roche, Indianapolis, IN) to remove trace amounts of residual genomic DNA. Genomic DNA was extracted from cell pellets by incubating the pellets in solutions containing 0.5 mg/mL Proteinase K, 2% SDS, and 20 mM Tris-HCl overnight at 55^o^C with shaking followed by phenol-chloroform extraction and ethanol precipitation. For 5-aza-2′-deoxycytidine (5-aza-dC) treatments, cells were seeded at 5×10^5^ cells per 100-mm dish 24 hr prior to the addition of 5-aza-dC (Sigma, St Louis, MO). Cells were treated with 5 µM 5-aza-dC for 48 hr before harvesting.

### MBD-isolated Genome Sequencing data analysis

Genome-wide DNA methylation data for HCT116 and DKO cells were previously obtained by performing MBD-isolated Genome Sequencing (MiGS) [21]. The raw sequencing reads can be downloaded from the NCBI Short Read Archive (SRA#SRP001414). The minimum number of sequencing reads indicating significant DNA methylation signals were determined as previously described. Annotated miRNAs (Hg18, NCBI Build 36.1) located within 500 bp of significant DNA methylation were identified as candidates for subsequent analyses.

### miRNA microarray analysis

Microarray-based miRNA expression profiling was performed using the Illumina human MicroRNA expression profiling V2 panel according to manufacturer's instructions. The human v2 miRNA panel contains 1,146 assays, for detection of ∼97% of the miRNAs described in the miRBase database (Sanger miRBase, v9.1, February 2007 release). Data processing was performed in GenomeStudio v2009.1 (Illumina, San Diego, CA). Triplicate experiments were performed for each cell line. Data from each experiment was normalized using the method described previously [37]. For the HCT116 versus DKO comparison, Welch's *t* test was performed with a *p*-value cutoff of 0.05 and multiple testing correction by false discovery rate (FDR). The microRNA array data (GEO# GSE26819) can be accessed from the Gene Expression Omnibus website.

### Bisulfite sequencing

1 µg of genomic DNA from each sample was bisulfite converted using the EpiTect kit (Qiagen, GmbH, Hilden) following manufacturer's protocol. PCR conditions and primer sequences are provided in Table S3. The PCR amplicons were gel-purified and subcloned into pCRII-TOPO vector (invitrogen, Carlsbad, CA). At least eight clones were randomly selected and sequenced on an ABI3730xl DNA analyzer to ascertain the methylation patterns of each locus.

### Detection of mature and primary miRNAs expression by quantitative RT-PCR

Expression of mature miRNAs was determined using QuantiTect SYBR Green RT-PCR kit (Qiagen, GmbH, Hilden) and was normalized to endogenous U6 snoRNA expression using the 2^-ΔΔCt^ method [38]. Briefly, 100 ng DNase-treated RNA from each sample was added to 10 µl of the 2× SYBR green PCR master mix, 0.2 µl RT-mix, 200 nM of each primer, and RNase-free water to a total of 20 µl. For primary miRNA expression, GAPDH was used as the normalization control. The expression of each primary miRNA relative to GAPDH was also determined using the 2^-ΔΔCt^ method. The primer sequences for detecting mature and primary miRNAs were designed as previously described [23], [39] and listed in Table S3. The mean of triplicate experiments was graphed with standard deviations represented as error bars.

### Detection of putative host gene expression by quantitative RT-PCR

Expression of putative host genes was determined using QuantiTect SYBR Green RT-PCR kit (Qiagen, GmbH, Hilden) and was normalized to GAPDH internally. The relative expression after 5-aza-dC treatment was calculated using the 2^-ΔΔCt^ method. The RNA was extracted in the same manner as stated above. The primer sequences are listed in Table S3. The mean of triplicate experiments was graphed with standard deviations represented as error bars.

### Ectopic expression of miRNAs

Over-expression of miR-941, miR-1237, and miR-1247 in HCT116, DKO, and DLD1 cells was achieved by transfecting the cells with 20 nM miRNA duplexes that mimicked the mature miR-941 (MSY0004984, Qiagen), miR-1237 (MSY0005592, Qiagen), and miR-1247 (MSY0005899, Qiagen). The AllStars Negative Control siRNA (1027281, Qiagen) was included as a negative control. Transfections were carried out using Lipfectamine™ 2000 transfection reagent (Invitrogen, Carlsbad, CA) according to the manufacturer's protocol. The transfection efficiency was evaluated by transfection with the AF488 fluorescently labeled AllStars Negative Control (1027284, Qiagen).

### Cell proliferation and viability assays

To assay for cell proliferation, at 24 hr post-transfection, cells were re-plated at 8×10^4^ cells/well for HCT116 and DLD1 in 24-well plates. At 96 hr after re-plating, 100 µl of CellTiter AQueous (Promega, Madison, WI) was added to the cells for 1 hour, and the absorbance was measured at 490 nm using a microplate reader according to the manufacturer's recommendations. At 48 and 96 hr after re-plating, cell numbers were counted using the trypan blue dye exclusion method. The mean of triplicate measurements was plotted with standard deviations represented as error bars. DKO cells were plated on Day 0 at 4.6×10^5^ cells/well in 12-well plates and transfected with miRNA mimic on Day 1. On Day 6 after plating, 100 µl of CellTiter AQueous (Promega, Madison, WI) was added to the cells for 1 hour, and the absorbance was measured at 490 nm using a microplate reader according to the manufacturer's recommendations. On Day 3 and Day 6 after plating, cell numbers were counted using the trypan blue dye exclusion method. The mean of triplicate measurements was plotted with standard deviations represented as error bars.

### BrdU incorporation assay

HCT116 cells transfected with miR-941 and miR-1247 mimics and the AllStars Negative Control were re-plated on coverslips at 24 hr post-transfection. At 48 hr after re-plating, cells were incubated with 10 µg/mL bromodeoxyuridine (BrdU) (Roche, Indianapolis, IN) for 4 hr. The cells were then fixed in 4% paraformaldehyde for 15 minutes, incubated with 2M HCl for 30 minutes to denature genomic DNA, and neutralized by 0.1M Na_2_B_4_O_7_ for 5 minutes. The cells were subsequently stained with mouse monoclonal anti-BrdU antibody (Roche, Indianapolis, IN) and AF488 anti-mouse antibody (Invitrogen, San Diego, CA). The cells were also stained with 4,6-diamidino-2-phenylindole (DAPI) (Sigma, St Louis, MO). The percentage of BrdU incorporation was determined by counting the number of BrdU-positive nuclei among DAPI-stained nuclei in 4 independent microscope fields. A minimum of 600 nuclei were counted for each experiment. The mean of triplicate experiments was plotted with standard deviation represented as error bars.

### Wound-healing assay

Cells were plated in 24-well plates and grown to confluence. The monolayer was wounded using the tip of a sterile 200 µl pipette. Cell debris was removed by washing twice with serum-free medium. Cells were then allowed to migrate into the denuded areas for 16 hr. Photographs were taken immediately after wounding (time 0 hr) and after 16 hr (time 16 hr) using the Leica DMI3000 B inverted microscope. The results were quantified as a percentage of the wound width closed by the cells at time 16 hr (wound width at time 0 hr minus the wound width at time 16 hr) divided by the wound width at time 0 hr. The experimental groups were normalized to the results of the mock transfected cells. The mean of six experiments was graphed with standard deviations represented as error bars.

### Migration assay

Transwell plates (24-well, 8-µm pore size, Costar, Corning, NY) were used to conduct the migration assay. 5×10^4^ cells suspended in McCoy's 5A media containing 1% FBS were added to each upper chamber while the lower chambers were filled with 500 µl of McCoy's 5A media containing 10% FBS. Cells were incubated at 37°C with 5% CO_2_ for 6 hr to allow trans-well migration. At 6 hr, cells on the upper surface of the filters were removed using cotton swabs. Cells that migrated to the lower surface of the filters were rinsed with PBS, fixed in methanol, and stained with Giemsa (Sigma, St Louis, MO). Photographs were taken using the Leica DMI3000 B inverted microscope. The percentage change in migration was determined by counting the numbers of cells that migrated to the lower surface of the filters using ImageJ software (Wayne Rasband, National institutes of Health, http://rsbweb.nih.gov/ij). The experiment was performed in triplicates, and nine separate microscopic fields were counted per membrane.

### Statistical analysis

Results were expressed as means ± standard deviation. Student *t* test was used to determine the overall significance between data points. A *P*-value of <0.05 was considered significant.

## Supporting Information

Figure S1 **Additional bisulfite sequencing validation.** Bisulfite sequencing was carried out for (**A**) miR-193a, (**B**) miR-9-3, and (**C**) miR-24-1 in parental HCT116 cells and DKO cells. The UCSC genome browser screen capture shows the DNA methylation signal in each sample. The rectangles mark the regions validated by bisulfite sequencing. Each circle represents a CpG dinucleotide. Black circles represent methylated cytosines while white circles represent unmethylated cytosines.
(TIF)

Figure S2 **Confirmation of miRNA microarray expression data by quantitative real-time RT-PCR analysis.** The fold changes in mature miRNA expression between DKO and HCT116 cells as measured by real-time RT-PCR (light blue) are compared to those measured by miRNA expression microarray (dark pink).
(TIF)

Figure S3 **DNA methylation patterns at putative host gene promoters.** UCSC genome browser screen captures of the promoter regions (±1000 bp from annotated transcription start site) for (**A**) *RPS6KA4*, putative host gene for miR-1237, (**B**) *C9orf3*, putative host gene for miR-24-1 and miR-27b, (**C**) *AB058779*, putative host gene for miR-602, (**D**) *ANKRD30BL*, putative host gene for miR-663b, and (**E**) *DNAJC5*, putative host gene for miR-941-1 and miR-941-3. The first track contains DNA methylation at these promoters in HCT116, and the second track contains DNA methylation in DKO cells as detected by MiGS.
(TIF)

Figure S4 **Functional analysis of miR-941, miR-1237, and miR-1247 in DLD1 cells.** (**A**) Growth curves and (**B**) MTT cell proliferation assays of mock transfected DLD1 cells and cells transfected with non-coding negative control miRNA (AllStars Neg.), miR-941, miR-1237, or miR-1247 mimics. (**C**) Wound-healing assay for mock transfected HCT116 cells and cells transfected with negative control (AllStars Neg.), miR-941, miR-1237, or miR-1247 mimics. Photographs were taken immediately after wounding and 16 hr later. The results were quantified and normalized to the mock transfected cells.
(TIF)

Figure S5 **Functional analysis of miR-941 and miR-1247 in DKO cells.** (**A**) Growth curves and (**B**) MTT cell proliferation assays of mock transfected DKO cells and DKO cells transfected with non-coding negative control miRNA (AllStars Neg.), miR-941, or miR-1247 mimics. (**D**) Transwell migration assays for mock transfected DKO cells and cells transfected with negative control (AllStars Neg.), miR-941, or miR-1247. Representative fields of invasive cells on the membrane are shown. The bar graph represents the average number of cells on the underside of the membranes in each treatment normalized to mock transfected cells.
(TIF)

Table S1 Summary of miRNAs methylated in HCT116 and DKO cells.
(DOC)

Table S2 Methylation and expression data for miRNAs.
(DOC)

Table S3 List of PCR primers.
(DOC)
