# Supplementary figures and images for: Identification and Functional Analysis of Epigenetically Silenced MicroRNAs in Colorectal Cancer Cells
Source: PLoS One. 2011 Jun 16;6(6):e20628. doi: 10.1371/journal.pone.0020628 (PMC3116843; doi:10.1371/journal.pone.0020628)

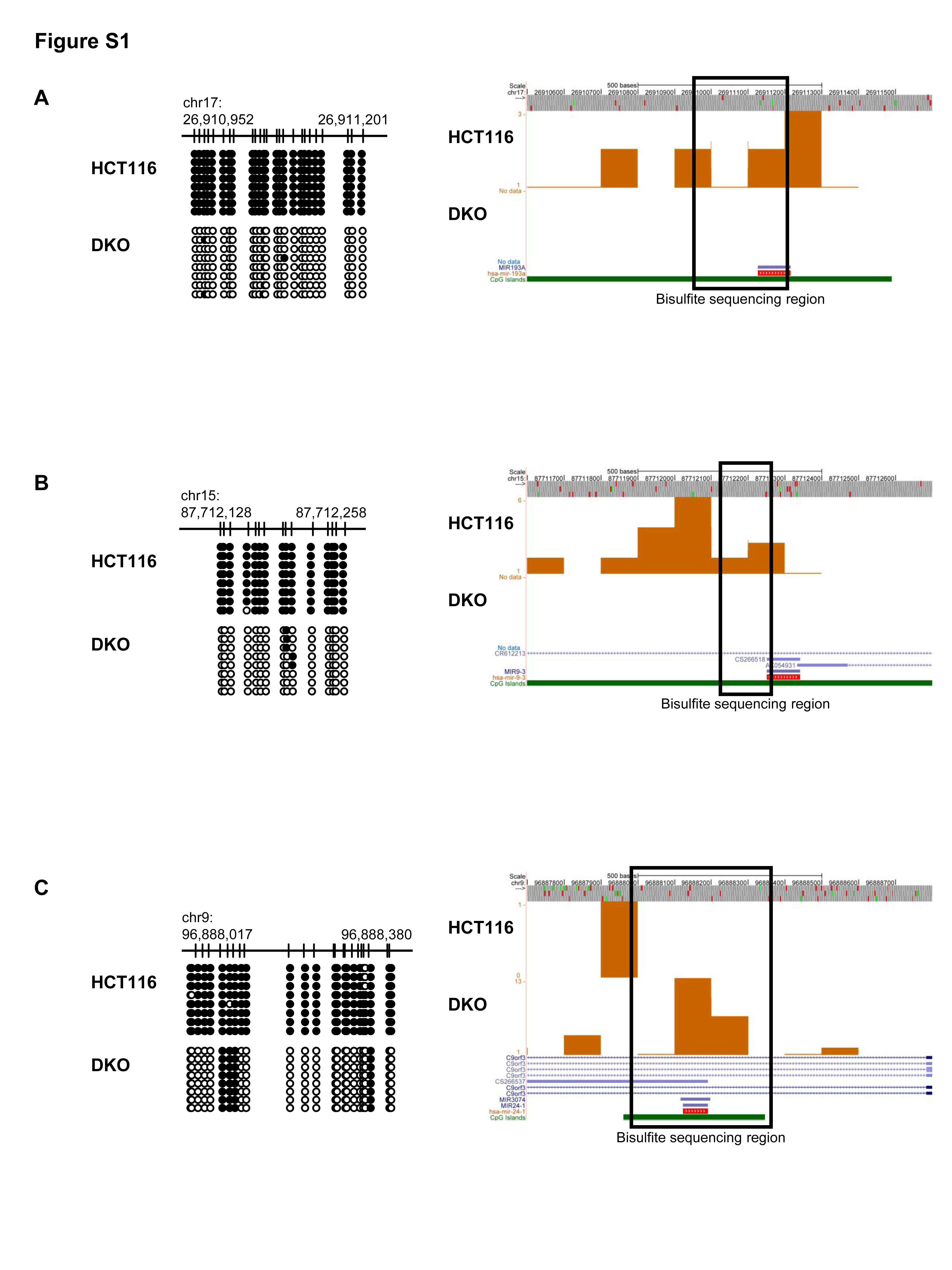

Supplement: Figure S1 — Additional bisulfite sequencing validation. Bisulfite sequencing was carried out for (A) miR-193a, (B) miR-9-3, and (C) miR-24-1 in parental HCT116 cells and DKO cells. The UCSC genome browser screen capture shows the DNA methylation signal in each sample. The rectangles mark the regions validated by bisulfite sequencing. Each circle represents a CpG dinucleotide. Black circles represent methylated cytosines while white circles represent unmethylated cytosines. (TIF) [file pone.0020628.s001.tif]

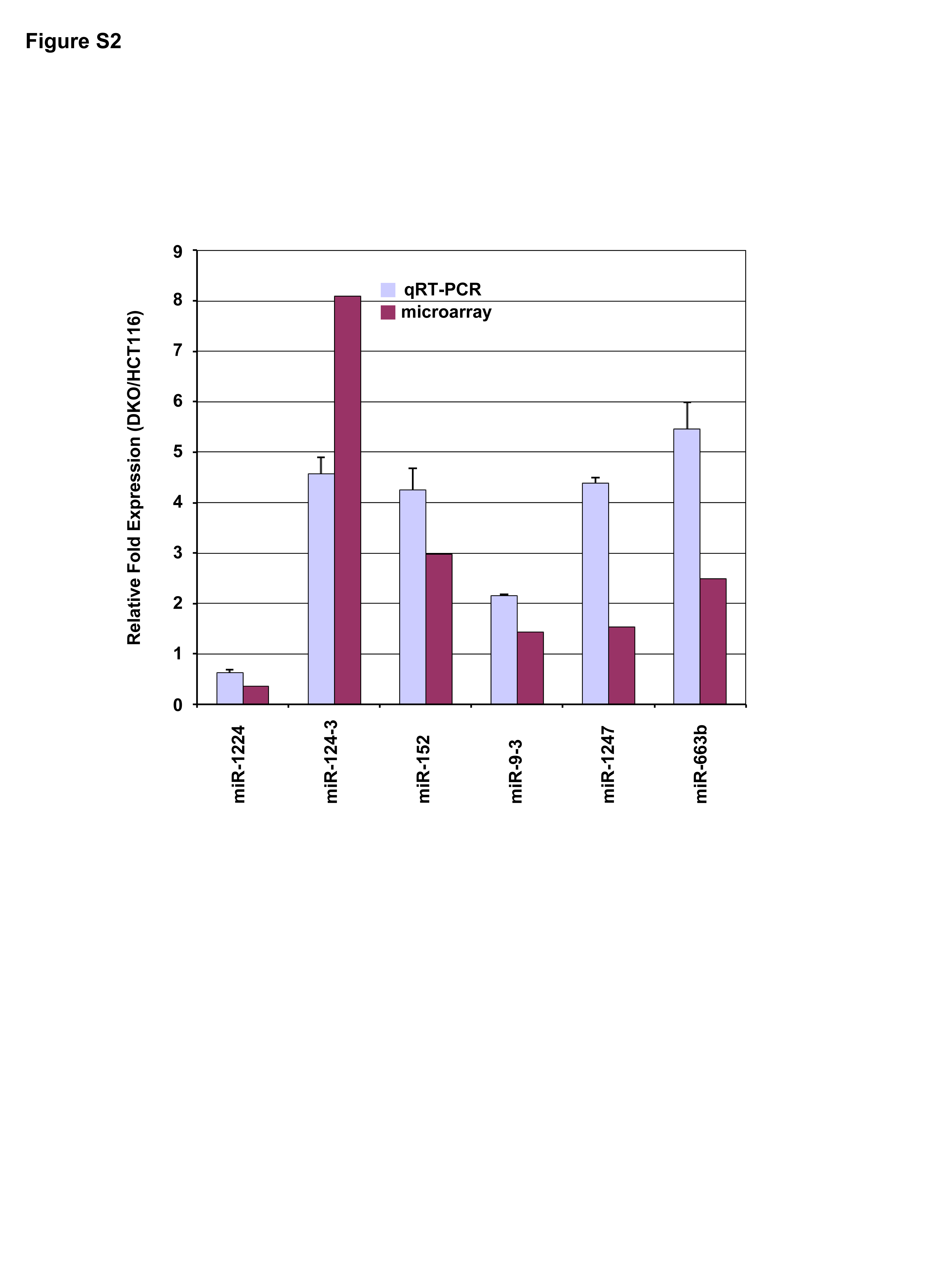

Supplement: Figure S2 — Confirmation of miRNA microarray expression data by quantitative real-time RT-PCR analysis. The fold changes in mature miRNA expression between DKO and HCT116 cells as measured by real-time RT-PCR (light blue) are compared to those measured by miRNA expression microarray (dark pink). (TIF) [file pone.0020628.s002.tif]

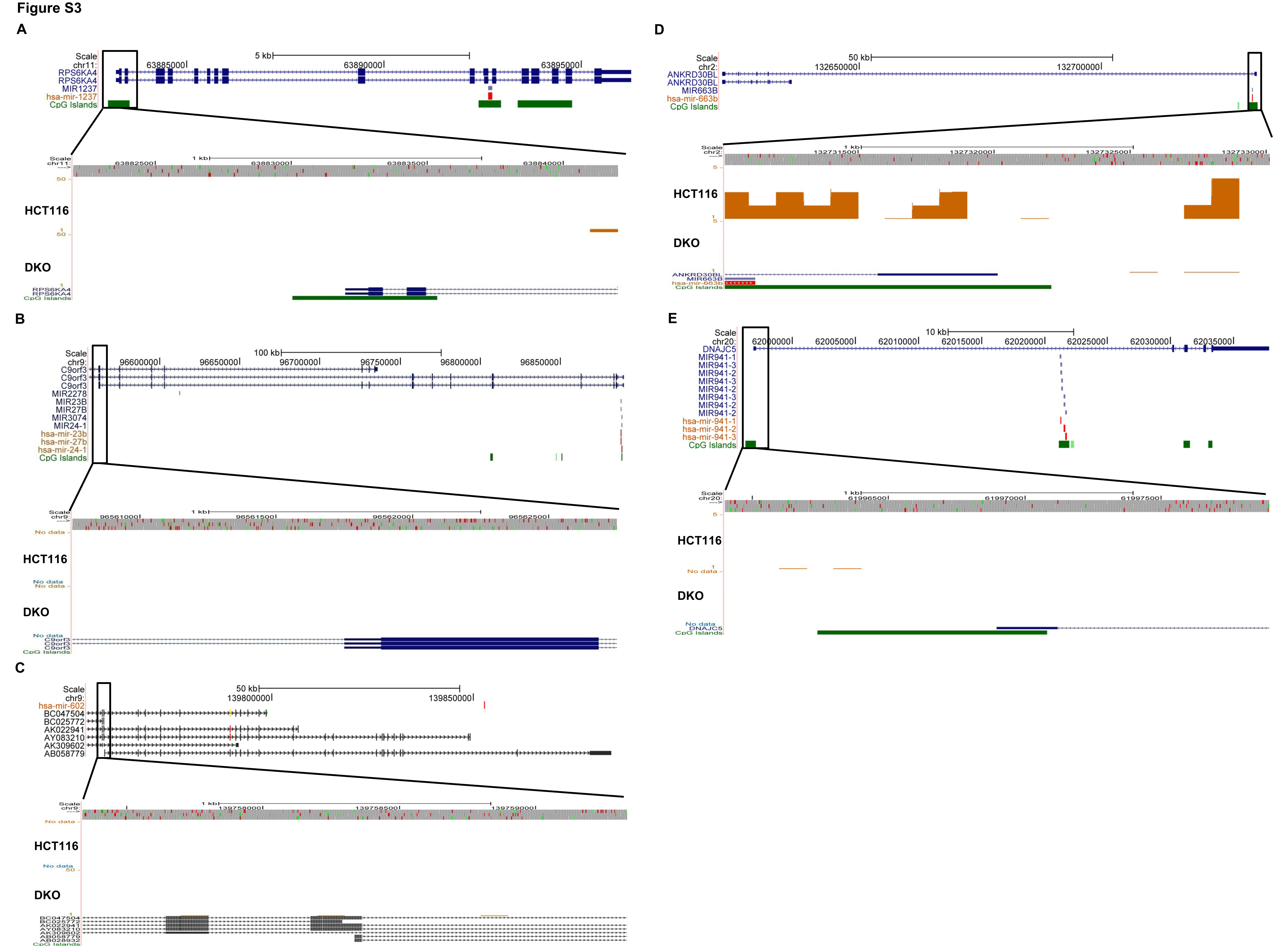

Supplement: Figure S3 — DNA methylation patterns at putative host gene promoters. UCSC genome browser screen captures of the promoter regions (±1000 bp from annotated transcription start site) for (A) RPS6KA4, putative host gene for miR-1237, (B) C9orf3, putative host gene for miR-24-1 and miR-27b, (C) AB058779, putative host gene for miR-602, (D) ANKRD30BL, putative host gene for miR-663b, and (E) DNAJC5, putative host gene for miR-941-1 and miR-941-3. The first track contains DNA methylation at these promoters in HCT116, and the second track contains DNA methylation in DKO cells as detected by MiGS. (TIF) [file pone.0020628.s003.tif]

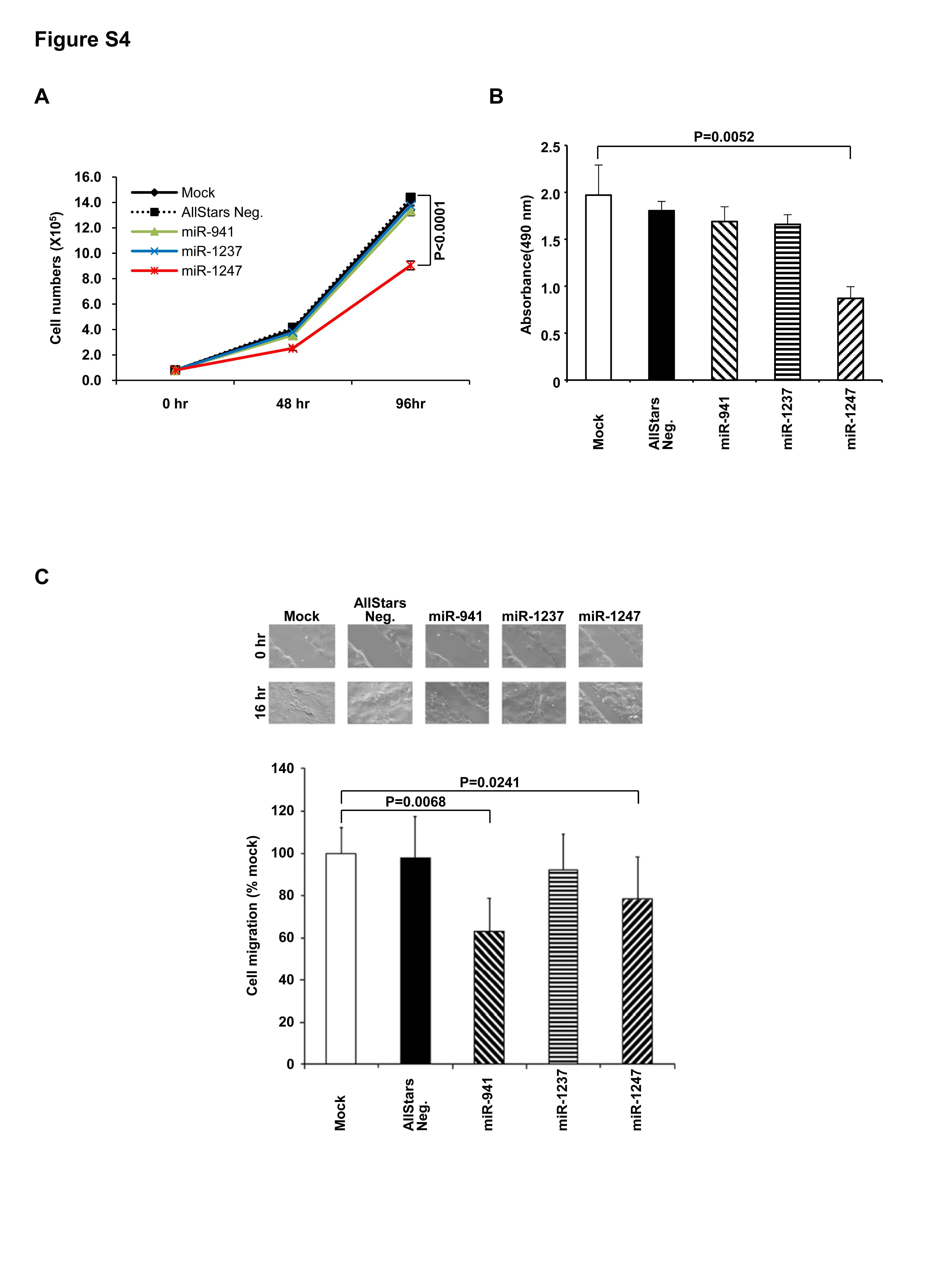

Supplement: Figure S4 — Functional analysis of miR-941, miR-1237, and miR-1247 in DLD1 cells. (A) Growth curves and (B) MTT cell proliferation assays of mock transfected DLD1 cells and cells transfected with non-coding negative control miRNA (AllStars Neg.), miR-941, miR-1237, or miR-1247 mimics. (C) Wound-healing assay for mock transfected HCT116 cells and cells transfected with negative control (AllStars Neg.), miR-941, miR-1237, or miR-1247 mimics. Photographs were taken immediately after wounding and 16 hr later. The results were quantified and normalized to the mock transfected cells. (TIF) [file pone.0020628.s004.tif]

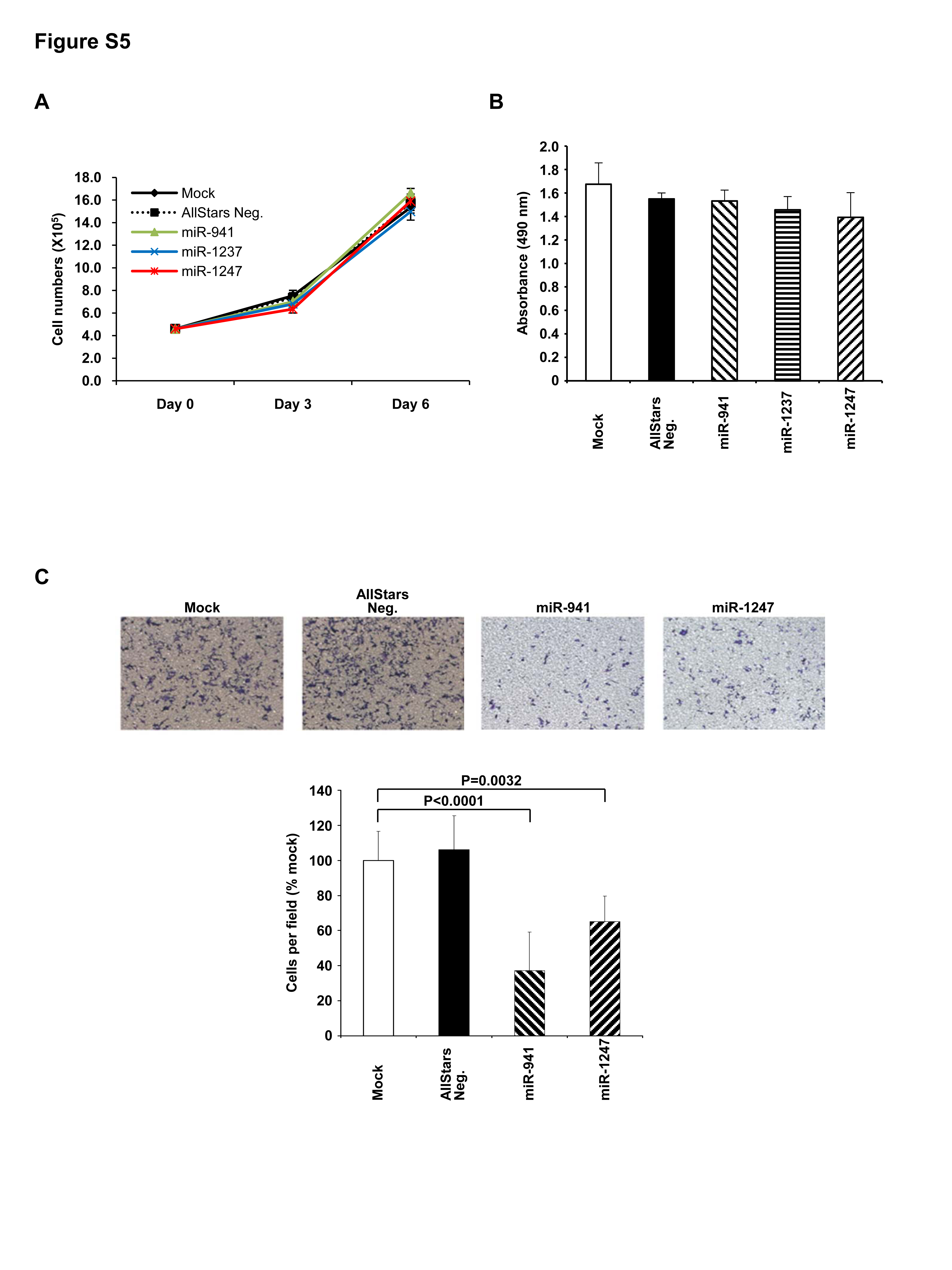

Supplement: Figure S5 — Functional analysis of miR-941 and miR-1247 in DKO cells. (A) Growth curves and (B) MTT cell proliferation assays of mock transfected DKO cells and DKO cells transfected with non-coding negative control miRNA (AllStars Neg.), miR-941, or miR-1247 mimics. (D) Transwell migration assays for mock transfected DKO cells and cells transfected with negative control (AllStars Neg.), miR-941, or miR-1247. Representative fields of invasive cells on the membrane are shown. The bar graph represents the average number of cells on the underside of the membranes in each treatment normalized to mock transfected cells. (TIF) [file pone.0020628.s005.tif]
