# Supplementary material for: Identification and Functional Analysis of Epigenetically Silenced MicroRNAs in Colorectal Cancer Cells
Source: PLoS One. 2011 Jun 16;6(6):e20628. doi: 10.1371/journal.pone.0020628 (PMC3116843; doi:10.1371/journal.pone.0020628)
Supplement: Table S1 — Summary of miRNAs methylated in HCT116 and DKO cells. (DOC) [file pone.0020628.s006.doc]

**Table S**1. Summary of miRNAs methylated in HCT116 and DKO cells.

| **miRNA** | **Methylation site** | **HCT116** | **DKO** | **CpG** | **Notes** |
| --- | --- | --- | --- | --- | --- |
| hsa-miR-1237 | chr11:63892300-63893200 | M^*^ | M | CpG | Consistent expression; **confirmed by 5-aza-dC** |
| hsa-miR-1247 | chr14:101095800-101097100 | M | U | CpG | Consistent expression; **confirmed by 5-aza-dC** |
| hsa-miR-1826 | chr16:33872500-33873600 | M | M | CpG | Consistent expression; **confirmed by 5-aza-dC** |
| hsa-miR-219-2 | chr9:130194300-130195400 | M | M | CpG | Consistent expression; **confirmed by 5-aza-dC** |
| hsa-miR-24-1 | chr9:96888200-96888600 | M | M | CpG | Consistent expression; **confirmed by 5-aza-dC** |
| hsa-miR-27b | chr9:96887100-96887300 | M | M |  | Consistent expression; **confirmed by 5-aza-dC** |
| hsa-miR-602 | chr9:139852700-139853000 | M | M |  | Consistent expression; **confirmed by 5-aza-dC** |
| hsa-miR-663b | chr2:132730500-132731700 | M | U | CpG | Consistent expression; **confirmed by 5-aza-dC** |
| hsa-miR-941-1 | chr20:62020900-62022300 | M | M | CpG | Consistent expression; **confirmed by 5-aza-dC** |
| hsa-miR-941-3 | chr20:62020900-62022300 | M | M | CpG | Consistent expression; **confirmed by 5-aza-dC** |
| hsa-miR-140 | chr16:68524100-68524300 | M | M |  | Consistent expression |
| hsa-miR-142 | chr17:53763200-53763500 | M | U |  | Consistent expression |
| hsa-miR-220b | chr19:6446400-6447200 | M | M | CpG | Consistent expression |
| hsa-miR-338 | chr17:76714700-76714900 | M | U |  | Consistent expression |
| hsa-miR-564 | chr3:44877900-44878200 | M | U | CpG | Consistent expression |
| hsa-miR-663 | chr20:26136600-26137500 | M | U | CpG | Consistent expression |
| hsa-miR-939 | chr8:145589600-145590600 | M | M | CpG | Consistent expression |
| hsa-miR-1234 | chr8:145595700-145596700 | M | M |  | Consistent expression |
| hsa-miR-1180 | chr17:19188400-19188600 | M | M | CpG |  |
| hsa-miR-1203 | chr17:43588600-43588800 | M | U |  |  |
| hsa-miR-1224 | chr3:185441600-185442400 | M | U | CpG | Negative control |
| hsa-miR-1225 | chr16:2079600-2080800 | M | U | CpG |  |
| hsa-miR-1226 | chr3:47865500-47866500 | M | U | CpG |  |
| hsa-miR-1227 | chr19:2184700-2184900 | M | U |  |  |
| hsa-miR-1228 | chr12:55874200-55874500 | M | U |  |  |
| hsa-miR-1229 | chr5:179157300-179158500 | M | U | CpG |  |
| hsa-miR-126 | chr9:138684300-138685100 | M | U | CpG |  |
| hsa-miR-1301 | chr2:25404600-25405000 | M | U |  |  |
| hsa-miR-149 | chr2:241043800-241044400 | M | U | CpG |  |
| hsa-miR-200b | chr1:1092300-1092500 | M | U |  |  |
| hsa-miR-203 | chr14:103653800-103654100 | M | U | CpG |  |
| hsa-miR-339 | chr7:1028600-1029500 | M | U | CpG |  |
| hsa-miR-33b | chr17:17657500-17658000 | M | U |  |  |
| hsa-miR-566 | chr3:50186200-50186400 | M | U |  |  |
| hsa-miR-572 | chr4:10979300-10980000 | M | U | CpG |  |
| hsa-miR-596 | chr8:1752300-1753400 | M | U | CpG |  |
| hsa-miR-637 | chr19:3911900-3912700 | M | U | CpG |  |
| hsa-miR-661 | chr8:145090800-145091100 | M | U | CpG |  |
| hsa-miR-671 | chr7:150565900-150567100 | M | U |  |  |
| hsa-miR-886 | chr5:135443700-135444300 | M | U | CpG |  |
| hsa-miR-935 | chr19:59177200-59177900 | M | M | CpG |  |
| hsa-miR-937 | chr8:144966800-144967400 | M | U | CpG |  |
| hsa-miR-943 | chr4:1957400-1957800 | M | U | CpG |  |
| hsa-miR-10a | chr17:44012400-44012600 | M | M |  | Known [12] |
| hsa-miR-124-1 | chr8:9797900-9798900 | M | U | CpG | Known [11] |
| hsa-miR-124-3 | chr20:61279700-61280900 | M | U | CpG | Known [11] |
| hsa-miR-127 | chr14:100419100-100419500 | M | U | CpG | Known [11] |
| hsa-miR-129-2 | chr11:43559000-43560000 | M | U | CpG | Known [11,16] |
| hsa-miR-137 | chr1:98283700-98284400 | M | U | CpG | Known [8,11] |
| hsa-miR-152 | chr17:43469300-43470200 | M | U | CpG | Known [14] |
| hsa-miR-193a | chr17:26910600-26911500 | M | U | CpG | Known [13] |
| hsa-miR-34b | chr11:110888300-110889100 | M | U | CpG | Known [9] |
| hsa-miR-34c | chr11:110888300-110889100 | M | U | CpG | Known [9] |
| hsa-miR-375 | chr2:219574300-219574800 | M | U | CpG | Known [40] |
| hsa-miR-9-1 | chr1:154656800-154657400 | M | U | CpG | Known [8] |
| hsa-miR-9-3 | chr15:87711700-87712900 | M | U | CpG | Known [15] |
| hsa-miR-410 | chr14:100601500-100602100 | M | U | CpG | Imprinted region |
| hsa-miR-431 | chr14:100417000-100417200 | M | M |  | Imprinted region |
| hsa-miR-433 | chr14:100417400-100418400 | M | M | CpG | Imprinted region |
| hsa-miR-543 | chr14:100567900-100568100 | M | U |  | Imprinted region |
| hsa-miR-675 | chr11:1974000-1974700 | M | U | CpG | Imprinted region |
| hsa-let-7a-3 | chr22:44886700-44887100 | M | U |  | No expression data |
| hsa-miR-1306 | chr22:18453200-18453900 | M | M | CpG | No expression data |
| hsa-miR-133a-2 | chr20:60572200-60572700 | M | U |  | No expression data |

Note: * M indicates DNA methylated, and U indicates DNA unmethylated.
