# Supplementary material for: Identification and Functional Analysis of Epigenetically Silenced MicroRNAs in Colorectal Cancer Cells
Source: PLoS One. 2011 Jun 16;6(6):e20628. doi: 10.1371/journal.pone.0020628 (PMC3116843; doi:10.1371/journal.pone.0020628)
Supplement: Table S2 — Methylation and expression data for miRNAs. (DOC) [file pone.0020628.s007.doc]

**Table S2**. Methylation and expression data for miRNAs.

| **miRNA** | **HCT116** | **DKO** | **Notes** | **Illumina Probe** | **Fold change (DKO/HCT116)** | **P value** |
| --- | --- | --- | --- | --- | --- | --- |
| hsa-miR-1237 | M | M | Consistent | hsa-miR-1237 | 0.65 | 2.77E-13 |
| hsa-miR-1247 | M | U | Consistent | hsa-miR-1247 | 1.54 | 0.00072618 |
| hsa-miR-1826 | M | M | Consistent | hsa-miR-1826 | 1.06 | 0.2121175 |
| hsa-miR-219-2 | M | M | Consistent | hsa-miR-219-2-3p | 1.04 | 0.8786814 |
| hsa-miR-24-1 | M | M | Consistent | hsa-miR-24 | 1.00 | 0.8852925 |
|  |  |  | Consistent | hsa-miR-24-1* | 0.58 | 0.000853733 |
| hsa-miR-27b | M | M | Consistent | hsa-miR-27b | 0.81 | 5.43E-06 |
|  |  |  | Consistent | hsa-miR-27b* | 0.52 | 4.33E-32 |
| hsa-miR-602 | M | M | Consistent | hsa-miR-602 | 0.77 | 0.2153792 |
| hsa-miR-663b | M | U | Consistent | hsa-miR-663b | 2.50 | 0.000673517 |
| hsa-miR-941-1 | M | M | Consistent | hsa-miR-941 | 1.29 | 0.000366146 |
| hsa-miR-941-3 | M | M | Consistent | hsa-miR-941 | 1.29 | 0.000366146 |
| hsa-miR-140 | M | M | Consistent | hsa-miR-140-3p | 1.15 | 0.000400981 |
|  |  |  | Consistent | hsa-miR-140-5p | 1.03 | 0.7554829 |
| hsa-miR-142 | M | U | Consistent | hsa-miR-142-3p | 9.88 | 3.68E-38 |
|  |  |  | Consistent | hsa-miR-142-5p | 5.62 | 2.19E-11 |
| hsa-miR-220b | M | M | Consistent | hsa-miR-220b | 0.85 | 0.3670451 |
| hsa-miR-338 | M | U | Consistent | hsa-miR-338-3p | 4.58 | 6.62E-09 |
|  |  |  | Consistent | hsa-miR-338-5p | 1.05 | 0.8575313 |
| hsa-miR-564 | M | U | Consistent | hsa-miR-564 | 0.73 | 0.000505613 |
| hsa-miR-663 | M | U | Consistent | hsa-miR-663 | 1.55 | 0.008360302 |
| hsa-miR-939 | M | M | Consistent | hsa-miR-939 | 0.65 | 4.31E-05 |
| hsa-miR-1234 | M | M | Consistent | hsa-miR-1234 | 1.09 | 0.3809845 |
| hsa-miR-1180 | M | M |  | hsa-miR-1180 | 1.52 | 3.90E-06 |
| hsa-miR-1203 | M | U |  | hsa-miR-1203 | 1.01 | 0.9711175 |
| hsa-miR-1224 | M | U | Negative control | hsa-miR-1224-3p | 0.48 | 1.79E-18 |
|  |  |  | Negative control | hsa-miR-1224-5p | 0.37 | 3.20E-20 |
| hsa-miR-1225 | M | U |  | hsa-miR-1225-3p | 0.62 | 0.02745783 |
|  |  |  |  | hsa-miR-1225-5p | 0.53 | 0.000245312 |
| hsa-miR-1226 | M | U |  | hsa-miR-1226 | 1.08 | 0.2538069 |
|  |  |  |  | hsa-miR-1226* | 0.83 | 0.06737991 |
| hsa-miR-1227 | M | U |  | hsa-miR-1227 | 0.83 | 0.00241976 |
| hsa-miR-1228 | M | U |  | hsa-miR-1228 | 1.06 | 0.639021 |
|  |  |  |  | hsa-miR-1228* | 1.32 | 0.001420552 |
| hsa-miR-1229 | M | U |  | hsa-miR-1229 | 0.82 | 3.76E-06 |
| hsa-miR-126 | M | U |  | hsa-miR-126 | 0.79 | 0.000354603 |
|  |  |  |  | hsa-miR-126* | 0.55 | 2.02E-29 |
| hsa-miR-1301 | M | U |  | hsa-miR-1301 | 1.24 | 4.00E-15 |
| hsa-miR-149 | M | U |  | hsa-miR-149 | 0.89 | 7.07E-05 |
|  |  |  |  | hsa-miR-149* | 1.09 | 0.7154878 |
| hsa-miR-200b | M | U |  | hsa-miR-200b | 1.05 | 0.07890978 |
|  |  |  |  | hsa-miR-200b* | 1.21 | 1.78E-13 |
| hsa-miR-203 | M | U |  | hsa-miR-203 | 0.26 | 7.36E-38 |
| hsa-miR-339 | M | U |  | hsa-miR-339-3p | 0.33 | 7.36E-38 |
|  |  |  |  | hsa-miR-339-5p | 0.76 | 2.26E-08 |
| hsa-miR-33b | M | U |  | hsa-miR-33b | 1.26 | 0.3511374 |
|  |  |  |  | hsa-miR-33b* | 0.30 | 7.36E-38 |
| hsa-miR-566 | M | U |  | hsa-miR-566 | 1.40 | 0.000473548 |
| hsa-miR-572 | M | U |  | hsa-miR-572 | 0.92 | 0.5953549 |
| hsa-miR-596 | M | U |  | hsa-miR-596 | 0.75 | 0.08081245 |
| hsa-miR-637 | M | U |  | hsa-miR-637 | 0.72 | 0.1968498 |
| hsa-miR-661 | M | U |  | hsa-miR-661 | 1.10 | 0.5069957 |
| hsa-miR-671 | M | U |  | hsa-miR-671-3p | 0.87 | 0.01836374 |
|  |  |  |  | hsa-miR-671-5p | 1.06 | 0.8083535 |
| hsa-miR-886 | M | U |  | hsa-miR-886-3p | 0.87 | 0.3305787 |
|  |  |  |  | hsa-miR-886-5p | 1.26 | 6.51E-11 |
| hsa-miR-935 | M | M |  | hsa-miR-935 | 2.46 | 3.68E-38 |
| hsa-miR-937 | M | U |  | hsa-miR-937 | 1.23 | 0.5227809 |
| hsa-miR-943 | M | U |  | hsa-miR-943 | 0.74 | 1.25E-06 |
| hsa-miR-10a | M | M | known | hsa-miR-10a | 0.30 | 7.36E-38 |
|  |  |  | known | hsa-miR-10a* | 0.31 | 7.36E-38 |
| hsa-miR-124-1 | M | U | known | hsa-miR-124 | 8.10 | 3.68E-38 |
|  |  |  | known | hsa-miR-124* | 1.18 | 0.6964674 |
| hsa-miR-124-3 | M | U | known | hsa-miR-124 | 8.10 | 3.68E-38 |
|  |  |  | known | hsa-miR-124* | 1.18 | 0.6964674 |
| hsa-miR-127 | M | U | known | hsa-miR-127-3p | 94.70 | 3.68E-38 |
| hsa-miR-129-2 | M | U | known | hsa-miR-129-3p | 11.41 | 3.68E-38 |
|  |  |  | known | hsa-miR-129-5p | 1.20 | 1.05E-08 |
| hsa-miR-137 | M | U | known | hsa-miR-137 | 0.74 | 0.04274783 |
| hsa-miR-152 | M | U | known | hsa-miR-152 | 2.97 | 3.68E-38 |
| hsa-miR-193a | M | U | known | hsa-miR-193a-3p | 1.71 | 3.46E-06 |
|  |  |  | known | hsa-miR-193a-5p | 3.14 | 3.68E-38 |
| hsa-miR-34b | M | U | known | hsa-miR-34b | 10.98 | 3.68E-38 |
|  |  |  | known | hsa-miR-34b* | 9.10 | 8.77E-09 |
| hsa-miR-34c | M | U | known | hsa-miR-34c-3p | 26.74 | 3.68E-38 |
|  |  |  | known | hsa-miR-34c-5p | 10.71 | 3.68E-38 |
| hsa-miR-375 | M | U | known | hsa-miR-375 | 1.76 | 3.68E-38 |
| hsa-miR-9-1 | M | U | known | hsa-miR-9 | 1.43 | 1.78E-15 |
|  |  |  | known | hsa-miR-9* | 0.57 | 6.37E-32 |
| hsa-miR-9-3 | M | U | known | hsa-miR-9 | 1.43 | 1.78E-15 |
|  |  |  | known | hsa-miR-9* | 0.57 | 6.37E-32 |
| hsa-miR-410 | M | U | Imprinted |  |  |  |
| hsa-miR-431 | M | M | Imprinted |  |  |  |
| hsa-miR-433 | M | M | Imprinted |  |  |  |
| hsa-miR-543 | M | U | Imprinted |  |  |  |
| hsa-miR-675 | M | U | Imprinted |  |  |  |
| hsa-let-7a-3 | M | U | No data |  |  |  |
| hsa-miR-1306 | M | M | No data |  |  |  |
| hsa-miR-133a-2 | M | U | No data |  |  |  |
