# Supplementary material for: Identification and Functional Analysis of Epigenetically Silenced MicroRNAs in Colorectal Cancer Cells
Source: PLoS One. 2011 Jun 16;6(6):e20628. doi: 10.1371/journal.pone.0020628 (PMC3116843; doi:10.1371/journal.pone.0020628)
Supplement: Table S3 — List of PCR primers. (DOC) [file pone.0020628.s008.doc]

**Table S3**. List of PCR primers

| **Gene** | **Primer name** | **Sequence (5'-3')** | **T_m_ (°C)** |
| --- | --- | --- | --- |
| U6 | U6-Primer-F | GCTTCGGCAGCACATATATAAAAT | 55 |
|  | U6-Primer-R | CGCTTCACGAATTTGCGTGTCAT |  |
| **Mature miRNA detection** | | | |
| Universal Forward | Universal -F | GTGCAGGGTCCGAGGT |  |
| mature miR-124-3 | miR-124-3(RT) | GTCGTATCCAGTGCAGGGTCCGAGGTATTCGCACTGGATACGACGGCATT | 53 |
|  | miR-124-3 (R) | GCCTAAGGCACGCGGTG |  |
| mature miR-922 | miR-922(RT) | GTCGTATCCAGTGCAGGGTCCGAGGTATTCGCACTGGATACGACGACGTA | 53 |
|  | miR-922(R) | TGA GCAGCAGAGAATAGGAC |  |
| mature miR-9-3 | miR-9-3(RT) | GTCGTATCCAGTGCAGGGTCCGAGGTATTCGCACTGGATACGACTCATAC | 55 |
|  | miR-9-3(R) | TGC GGT CTT TGG TTA TCT AGC |  |
| mature miR-1224 | miR-1224 (RT) | GTCGTATCCAGTGCAGGGTCCGAGGTATTCGCACTGGATACGAC CCACCT | 55 |
|  | miR-1224(R) | GTGAGGACTCGGGAGGT |  |
| mature miR-152 | miR-152 (RT) | GTCGTATCCAGTGCAGGGTCCGAGGTATTCGCACTGGATACGACCCAAGT | 55 |
|  | miR-152(R) | GCC TCA GTG CAT GAC AGA AC |  |
| mature miR-663b | miR-663b(RT) | GTCGTATCCAGTGCAGGGTCCGAGGTATTCGCACTGGATACGACCCTCAG | 53 |
|  | miR-663b(R) | GCCGGTGGCCCGGCCGTGC |  |
| mature miR-1237 | miR-1237(RT) | GTCGTATCCAGTGCAGGGTCCGAGGTATTCGCACTGGATACGACCTGGGG | 55 |
|  | miR-1237 (R) | GTATCCTTCTGCTCCGTC |  |
| mature miR-1247 | miR-1247(RT) | GTCGTATCCAGTGCAGGGTCCGAGGTATTCGCACTGGATACGACTCCGGGG | 55 |
|  | miR-1247 (R) | GATACCCGTCCCGTTCGT |  |
| mature miR-941 | miR-941(RT) | GTCGTATCCAGTGCAGGGTCCGAGGTATTCGCACTGGATACGACGCACAT | 55 |
|  | miR-941 (R) | AAGCACCCGGCTGTGTGCAC |  |
| **Primary miRNA detection primers** | | | |
| Pri-miR-1224 | pri-miR-1224-F | CGCCGTCACAAACTCTGTAG | 55 |
|  | pri-miR-1224-R | GGGAGAAGCGAGCTGAAAC |  |
| Pri-miR-9-3 | pri-miR-9-3-F | TGGGATAAGGTCAGAGATAGGA | 60 |
|  | pri-miR-9-3-R | AAGCCAGTGAGAATGGGATG |  |
| Pri-miR-193a | pri-miR-193a-F | ACCCCGAACTCCGAGGAT | 55 |
|  | pri-miR-193a-R | TGGGACTTTGTAGGCCAGTT |  |
| Pri-miR-375 | pri-miR-375-F | AGACCAGGACCAGGAGATCA | 60 |
|  | pri-miR-375-R | AACGAACAAAACGCTCAGGT |  |
| Pri-miR-564 | pri-miR-564-F | GTGTCAGCAGGCAACATGG | 55 |
|  | pri-miR-564-R | GGCGCACAGACAGTTGAATA |  |
| Pri-miR-663b | pri-miR-663b-F | GGAGAAACCTCAGGCATGG | 55 |
|  | pri-miR-663b-R | CGTGAGGCAGGTCTTGGT |  |
| Pri-miR-663 | pri-miR-663-F | CGGATCTCGAGGGTGCTTAT | 55 |
|  | pri-miR-663-R | ACTTTCCACCGCAGCCTTC |  |
| Pri-miR-1826 | pri-miR-1826-F | GGATCACTGGGCTTCTGTGT | 55 |
|  | pri-miR-1826-R | CTGCAATTGCGTTCGAAGT |  |
| Pri-miR-142 | pri-miR-142-F | AGGGAGGTAGAGGAGGCAAG | 60 |
|  | pri-miR-142-R | CTCCTGACTCCTGCTCCAAG |  |
| Pri-miR-140 | pri-miR-140-F | CGTGGATGGATGTTCCTTTT | 55 |
|  | pri-miR-140-R | TGGCAGGACACAGAGAGAGA |  |
| Pri-miR-219-2 | pri-miR-219-2-F | CACAGATGTCCAGCCACAAT | 55 |
|  | pri-miR-219-2-R | TTTTAGGAGCGAAGGGGAAC |  |
| Pri-miR-338 | pri-miR-338-F | GAAGAAGTGGCGAAGGACAC | 55 |
|  | pri-miR-338-R | TGCCCTCTTCAACAAAATCA |  |
| Pri-miR-1247 | pri-miR-1247-F | AACGCTCAGCACCCATTTAC | 55 |
|  | pri-miR-1247-R | CGGACGTTGCTCTCTACCC |  |
| Pri-miR-1237 | pri-miR-1237-F | GGAGTTCGCAGTCAAGATCC | 55 |
|  | pri-miR-1237-R | ACGGAGCAGAAGGAGTTACG |  |
| Pri-miR-602 | pri-miR-602-F | CAAAAACTCAACGCACGAGA | 55 |
|  | pri-miR-602-R | CCTGACAGGAGACGCACTC |  |
| Pri-miR-939 | pri-miR-939-F | TGGGTTTTCTGGGTATGTGG | 55 |
|  | pri-miR-939-R | TGGAGGAAGCACTCACCTTC |  |
| Pri-miR-220b | pri-miR-220b-F | TGTCGTAGAGTGCCTCGTTG | 55 |
|  | pri-miR-220b-R | CGCATCATGAACACCTTCAG |  |
| Pri-miR-24-1 | pri-miR-24-1-F | CGGTGCCTACTGAGCTGAT | 55 |
|  | pri-miR-24-1-R | CCTCGGGCACTTACAGACAC |  |
| Pri-miR-27b | pri-miR-27b-F | GGAAACAAAAGAAGCCACCA | 55 |
|  | pri-miR-27b-R | ACAAAGCGGAAACCAATCAC |  |
| Pri-miR-941-1 | pri-miR-941-1-F | TACCACTGAGTCCCCAGCTC | 60 |
|  | pri-miR-941-1-R | ACACGTCGGTGCCATCAC |  |
| Pri-miR-941-3 | pri-miR-941-3-F | GGAGTTCGCAGTCAAGATCC | 60 |
|  | pri-miR-941-3-R | ACGGAGCAGAAGGAGTTACG |  |
| **Bisulfite sequencing primers** | | | |
| miR-941-3 | BSP-941-3-F | TTAGTAGGGAGTAGGGTAGGGTGTT | 55 |
|  | BSP-941-3-R | ATTAAAAACAAACAAAAAAAATCAC |  |
| miR-1237 | BSP-1237-F | TGTATTAGAATGTAATTGGAATATTTTTTA | 55 |
|  | BSP-1237-R | ACACACAAAAAAACTACCCTAACC |  |
| miR-1247 | BSP-1247-F | TTTTGGTTTTGTAGTTGGTTTTTTT | 55 |
|  | BSP-1247-R | CTTAAACTACAATATCCCCCAAAAC |  |
| miR-9-3 | BSP-9-3-F | GTTTGTTTATTTTTTTTGGTTTTT | 55 |
|  | BSP-9-3-R | GTTGTAGGAGGTATTAGGTTTTTTT |  |
| miR-24-1 | BSP-24-1-F | TTTTTTGTATTTGTAGTTTAGGTTTT | 55 |
|  | BSP-24-1-R | TACCAACAAAATTTTCCAAATCTAC |  |
| miR-193a | BSP-193a -F | GTAATTTTTGGAGGGTTGGGTTTG | 55 |
|  | BSP-193a -R | TAAAACTTTATAAACCAATTAATCC |  |
| **Host gene detection primers** | | | |
| AB058779 | AB058779-F | CGACGTTCGAGAGGAAGATT | 58 |
|  | AB058779-R | TCCCCCTCCACCATAAGAGT |  |
| RPS6KA4 | RPS6KA4F | CACAAGCTCGGCATCATTTA | 55 |
|  | RPS6KA4R | GAAGGTCCGCTCTTTCTCCT |  |
| DNAJC5 | DNAJC5F | ACGCCACAAAAAGGAACATC | 58 |
|  | DNAJC5R | TGGACAGCACGAAGTAGGTG |  |
| C9orf3 | C9orf3-F | CCAAGGAGCAGATGGATAGG | 55 |
|  | C9orf3-R | ATGTCCAGGGTCAGTTCGAG |  |
| ANKRD30BL | ANKRD30BL-F | AGAGGACCCTCCTCCACTTC | 58 |
|  | ANKRD30BL-R | TTGCCACCACTGACAAATTC |  |
